# Comparative Analysis of Broiler Housing Systems: Implications for Production and Wellbeing

**DOI:** 10.3390/ani14111665

**Published:** 2024-06-02

**Authors:** Hen Honig, Amit Haron, Liran Plitman, Dmitry Lokshtanov, Dmitry Shinder, Sagit Nagar, Tamir Goshen, Shelly Druyan

**Affiliations:** 1 Veterinary Services, Ministry of Agriculture and Rural Affairs, Beit Dagan 5025001, Israel; henh@moag.gov.il (H.H.);; 2 Institute of Animal Science, Agricultural Research Organization, Volcani Center, HaMaccabim Road, Rishon Le Zion 7528809, Israel

**Keywords:** broilers, colony cages, welfare, production performance, thermal regulation, leg deformities

## Abstract

**Simple Summary:**

In this study, we compared two different methods of raising broiler chickens: modern colony cages and traditional floor systems. We aimed to understand how each system impacts both the production and wellbeing of the chickens. We discovered that, while colony cages excel in maintaining optimal environmental conditions and promoting rapid growth, they also create challenges, such as leg deformities and difficulty standing. Conversely, traditional floor systems present issues such as heat stress, affecting broiler growth. Our findings emphasize the need to achieve a balance between chicken welfare and production objectives in poultry farming. By recognizing these differences, we can strive to enhance broiler rearing practices, ensuring the birds’ wellbeing while still meeting the consumer demand for poultry products.

**Abstract:**

This study compares the effects of modern colony cage systems and traditional floor systems on the production and welfare of broiler chickens. Through two trials spanning 35 days each, we evaluated various physiological parameters, including growth performance, bone health, stress responses, and meat quality. Colony cages demonstrated superior thermal regulation and growth performance compared to traditional floor systems, but also exhibited higher frequencies of leg deformity and reduced standing ability. Conversely, the broilers in traditional floor systems experienced heat stress-related challenges, impacting the meat quality. Our findings underscore the need to balance productivity with animal welfare in broiler farming practices. By understanding the distinct impacts of different housing systems, we can work towards improving broiler rearing methods to ensure optimal welfare and production outcomes.

## 1. Introduction

The broiler industry is the most intensive of all food animal production industries, and the number of chickens raised for meat is constantly on the rise, aiming to satisfy the demands of the growing human population. The number of chickens slaughtered increases annually, with over 73 billion chickens slaughtered worldwide by 2021 [1]. To meet this ever-growing need, the broiler industry aims to achieve high performance and efficiency in terms of weight, feed conversion, and meat yields while maintaining broiler welfare.

The EU Directive (2007/43/EC) mandates that all broilers have permanent access to dry and friable litter [2]. Accordingly, broilers in the EU are mainly raised in loose housing systems on concrete floors with deep litter, which consists of organic materials such as wood shavings, rice husks, and peanut shells [3,4,5,6]. Providing litter is essential to allow broilers to engage in their natural behaviors, including scratching, pecking, foraging, and dustbathing [7], which contribute to maintaining healthy plumage and removing ectoparasites [8].

However, this method has its drawbacks, such as increased litter management costs and the potential for poor air and litter quality due to elevated ammonia levels [9,10,11,12]. These conditions can lead to various health issues in broilers, including upper respiratory diseases, the spread of enteric diseases, foot pad dermatitis, and physical foot damage [13,14]. Although traditional litter-based systems are widely used in broiler production, they bring challenges such as direct contact with fecal uric acid and the requirement for more floor space [15,16]. Moreover, meticulous litter management is crucial in maintaining optimal litter quality and controlling indoor ammonia levels. Suboptimal litter and indoor air quality can adversely affect broiler welfare, performance, carcass yields, and meat quality [9,10,11,12]. An alternative to floor rearing is a multi-tier colony or battery cages with perforated or plastic-coated wire flooring without any supplementary bedding material. In broiler production, multi-tier colony cage systems have gained popularity in recent years due to limited land space, providing increased efficiency per poultry house and decreased production costs, reducing the fixed expenses per broiler [17,18,19]. Multi-tier cage systems, however, are not widely adopted due to criticism in terms of regulation in the Western part of the world, animal welfare, and behavioral restrictions, as has also been the case concerning egg production [20].

An evaluation of the effects of both multi-tier colony cages and deep litter housing systems on broilers’ welfare (Welfare Quality^®^ assessment protocol (WQP)) found both systems to score highly in almost all measurements [21]. Nevertheless, some measurements that could indicate a possible negative effect of the housing system on broiler behavior were performed only partially and, therefore, could not provide a full picture of the impact of multi-tier colony cages on the welfare of broilers [21]. The Five Domains Model is now commonly accepted as a framework to assess animal welfare, including both physical/functional (‘nutrition’, ‘environment’, ‘health’, and ‘behavior’) and mental (‘mental state’) domains, interacting with each other [22].

While the majority of the research performed to date has focused on production performance or welfare, fewer studies have been performed using a holistic approach to comprehensively assess multi-tier colony cages and compare them to the traditional deep litter broiler houses. The current study was carried out to assess the physiological effects of the housing system (deep litter vs. multi-tier colony cages) on selected production and welfare traits of commercial Ross 308 broilers.

## 2. Materials and Methods

The study was approved by the Agricultural Research Organization (ARO) Institutional Animal Care and Use Committee and was carried out in compliance with the current laws governing biological research in Israel (approval number: 927/21 IL).

### 2.1. Housing and Experimental Procedure

This study was conducted on two commercial broiler farms located 5 km apart, each with distinct characteristics, as outlined in Table S1.

Two commercial broiler farms were utilized for this experiment.

A. The traditional Israeli broiler farm (TF) comprised five houses, featuring open-sided structures with wire mesh fences and wood shaving litter covering the concrete floor. Each house provided usable space amounting to 1250 m^2^ and was equipped with 288 feeders and 1280 nipple drinkers. With a capacity of up to 22 thousand broilers per house (according to the regulated density per meter), two out of the five houses were selected for this study (TF1 and 2).
B. The modern multi-tier colony cage housing system (CCS) comprised three houses. The cages were constructed with zinc–aluminum-coated wire, side-opening grills, soft flexible plastic mesh flooring, and a polypropylene conveyor belt underneath. The system featured six rows of 5-tier cages, totaling 50 cages per row. Each cage offered usable space amounting to 3.7 m^2^ and was furnished with 2 feeders and 12 nipple drinkers. The floor was divided into pivoting sections to facilitate broilers’ removal to the conveyor belt during marketing, as well as the collection and removal of broiler droppings from the house in each row.

Two consecutive experiments (Trials 1 and 2), each lasting 35 days, were conducted to align with typical market ages for broilers in Israel, targeting a marketing weight of 2 to 2.5 kg. Trial 1 was conducted during the summer season and Trial 2 during the autumn. Ross 308 broiler chickens were sourced from the same parent flock. For each experiment, hatching broiler chicks of mixed sex were obtained from the same hatchery (Ma’anait LTD). Initially, the chicks were placed in TF (houses 1 and 2), followed by the placement of the hatching chicks in the CCS farm two days later (house 3). It is important to note that the transportation and broiler growth management were conducted by the same organization throughout both trials.

### 2.2. Density and Selected Individual Birds

In both trials, the chicks were delivered to the farms on the day of hatching and weighed upon arrival. From this cohort, 600 individual birds (300 per farm) were selected as the experimental population based on a mean body weight of ± 2 g, with each bird being assigned a unique serial number.

In Trial 1, the chicks originated from a 48-week-old (775) breeder flock, with an average chick weight of 46 ± 2 g. In Trial 2, the chicks originated from a 32-week-old (775) breeder flock, with an average chick weight of 40 ± 2 g.

The experimental groups were established at the farms according to predefined settings (see Table S1). The stocking density in both farms was set at 15.75 chicks per square meter.

At the TF farm, two houses (houses 1 and 2) were selected for both trials to avoid confounding effects. Initially, the density in each house was set at 12 chicks per square meter. To achieve the desired density, a separate pen was constructed within each house to accommodate 1200 birds, resulting in a density of 15.75 chicks per square meter. The 300 selected and tagged chicks were evenly divided between the two houses (150 selected and tagged chicks in each house).

At the CCS farm, 30 random cages were selected from a total of 1500 cages, each representing a unique row and floor combination (see Table S2). The 300 selected broilers were then randomly distributed among these 30 cages, with 10 chicks allocated to each cage. The total number of broilers per cage was 57, resulting in a density of 15.75 chicks per square meter. The sex of each bird was determined when secondary sex markers began to appear.

### 2.3. Management Protocol

#### 2.3.1. Nutrition and Lighting Program

The diet was formulated based on established management guidelines, with both water and feed provided ad libitum. The feeding program comprised pre-starter feed from day 0 to day 10, starter feed from day 11 to day 18, grower feed from day 19 to day 28, and finisher feed from day 29 to the end. The contents of CP (%) and energy (cal/kg of ME) in these four diets were as follows: 21.5 and 3030, 20.3 and 3050, 18.5 and 3110, and 18.0 and 3175, respectively. The house temperature was regulated according to the recommended parameters, starting from 34 °C on the day of hatching and gradually decreasing to 21 °C from day 21 onwards. The relative humidity (RH) was maintained at 56%. The photoperiod followed a specific schedule: 23 h of light and 1 h of darkness from days 0 to 7, 18 h of light and 6 h of darkness from days 7 to 21, 19 h of light and 5 h of darkness from days 21 to 28, and, finally, 20 h of light and 4 h of darkness from day 28 until the end of the trial. Additionally, a caretaker walked through each house daily to familiarize the birds with the regular presence of humans.

#### 2.3.2. Environmental Factors

The air temperature (Temp) and relative humidity (RH) were monitored using data loggers (D-LogMate-2TCK, 2 Channels, K Thermocouple Data-Logger, MRC Laboratory Equipment Ltd., Holon, IL, USA) installed in both the TF and CCS houses. In the TF houses, two data loggers were placed in each experimental pen, while in the CCS houses, individual data loggers were positioned in each selected cage, as detailed in Table S2. Measurements were taken every hour, resulting in four measurements per hour per data logger. The average Temp and RH values were then calculated based on these measurements for each pen or cage per hour.

#### 2.3.3. Production Performance

Body Weight—The initial body weights of randomly selected chicks were recorded immediately after transport (150 for TF1 and TF2 and 300 for CCS in both trials) using a digital scale (Kern DE 6K1D, KERN & SOHN GmbH, Balingen, Germany). Subsequent weight measurements were taken on days 0, 7, 14, 21, 28, and 34. Weight gain was calculated as the difference between the current body weight and the weight at the previous measurement.

Body Temperature Measurements—The broilers’ body temperatures were measured weekly using a digital thermometer (Super Speed Digital Thermometer; Pro-Care Measure Technology Co., San Chung City, Taipei, Taiwan) with an accuracy of ±0.1 °C. Temperature readings were taken by inserting the thermometer 1.5 cm into the cloaca for all selected individual birds (150 for TF1 and TF2 and 300 for CCS in both trials).

Meat Yield and Quality—At 35 days of age, a slaughter test was conducted on the selected broilers for bleeding on each farm (the rest of the birds were shipped to the slaughterhouse). Prior to slaughter, the feed was removed for 12 h. Each bird was weighed and labeled with a tag number on its legs before processing. During processing, the breast muscles were examined for muscle myopathies (wooden breast—WB, spaghetti muscle—MS, and green muscle disease—GMD), blisters, and cuts. The dissected breast muscles, abdominal fat pad, heart, and liver were removed and weighed, and their weights were calculated relative to the live body weight. Breast fillets were stored at 4 °C for 72 h and reweighed to determine the drip loss [(breast weight at dissection—breast weight after 72 h)/breast weight at dissection)].

Broiler Wellbeing: Blood Hormone Parameters—Starting from day 7, approximately 1 mL of blood was drawn once a week from the jugular vein in 75 random birds in Trial 1 and from 50 in Trial 2 from each TF house (a total of 150 or 100 birds out of 300 from Trials 1 and 2, respectively) and 5 chicks per cage (5 out of 10 selected birds from each cage, totaling 150 chicks in both trials) in the CCS. Blood was collected into heparinized 25 G and later 23 G syringes. The thyroxin (T4) and triiodothyronine (T3) concentrations were determined using commercial radioimmunoassay kits (Diagnostic Products Corporation). The plasma corticosterone concentrations were measured using a DetectX Enzyme immunoassay kit from Arbor Assays (Ann Arbor, MI, USA). The plasma samples were diluted 1:100, and the immunoassay was performed according to the kit instructions. The corticosterone concentrations were calculated from a standard curve and expressed as nanograms of corticosterone per milliliter of plasma.

The hormone level indicators were only tested in the birds in the first trial, as, at the conclusion of the second trial, following the findings of the gross pathology and LTL, the broiler growth management method using the CCS cages was discontinued, as will be explained in the Discussion.

Standing Ability and Leg Deformities: At 34 days of age, the broilers’ leg standing ability, footpads, and hock burn lesions were visually assessed by two separate research technicians. The leg standing ability was evaluated using the following scoring system: 0 (normal, no detectable abnormality); 1 (bird is standing but has a defect in one leg); 2 (bird can stand but has definite, identifiable defects in both legs); 3 (obvious defect in one leg that affects standing); 4 (severe defect, bird cannot stand on both legs). Hock burn lesions and footpad lesions were scored according to Butterworth (2013) [23].

Skin Integrity: Before the slaughter process, the presence or absence of skin tears and superficial and deep scratches on the skin surface of the breast, back, shoulder, pygostyle, and cloaca area was assessed. Scoring was conducted based on predefined criteria.

Ash Content: In the first trial, the right tibiae were removed from the carcasses immediately after slaughter and stored at −20 °C. Upon thawing, the tibiae were stripped of muscle and weighed. Subsequently, they were boiled in distilled water for 5 min to facilitate the removal of any remaining muscle and connective tissue, oven-dried overnight at 70 °C, cooled in a desiccator, weighed, and ashed for 12 h in a furnace at 600 °C. The ash content was expressed as a percentage of the dry bone weight.

Latency-to-Lie Tests (LTL): In the second trial, selected bled broilers underwent LTL tests, conducted at the end of the trial. The LTL test was adapted from previous methodologies [24,25]. Each bird underwent the LTL individually. The birds were placed in a plastic storage tub measuring 0.8 m long and 0.5 m wide. Warm tepid water (32 °C) was poured into the tub to a depth of 30 mm. The test began when each bird stood after the introduction of the water. If a bird did not stand initially, it was gently encouraged to do so, and the test commenced. Birds failing to stand were classified as lame. The test was concluded when the bird sat for 3 s or longer, with the stopwatch stopped, and the LTL time was recorded. If the bird did not sit after 10 min, the test ended and an LTL time of 10 min was recorded.

### 2.4. Statistical Analysis

Due to the interaction between the seasons (Trial 1 or 2) and houses, the differences between the houses (CCS, TF1, and TF2) were assessed separately for each season (Trials 1 and 2), and the data were analyzed using the following two-way ANOVA model:

Y=μ+House+Sex+House×Sex+e 

House (CCS, TF1, and TF2) and sex (male or female) were the main fixed effects, and their interaction (House x Sex) was also studied.

The Tukey–Kramer HSD test was used for the post hoc testing of the differences between the LS means.

The analysis of mortality as the proportion of ‘1’ alive versus ‘0’ dead individual birds, as well as breast normality as the proportion of ‘1’ normal versus ‘0’ diagnosed within each house, was tested via a chi-square test.

## 3. Results

### 3.1. Indoor Environment

**Figure 1:**
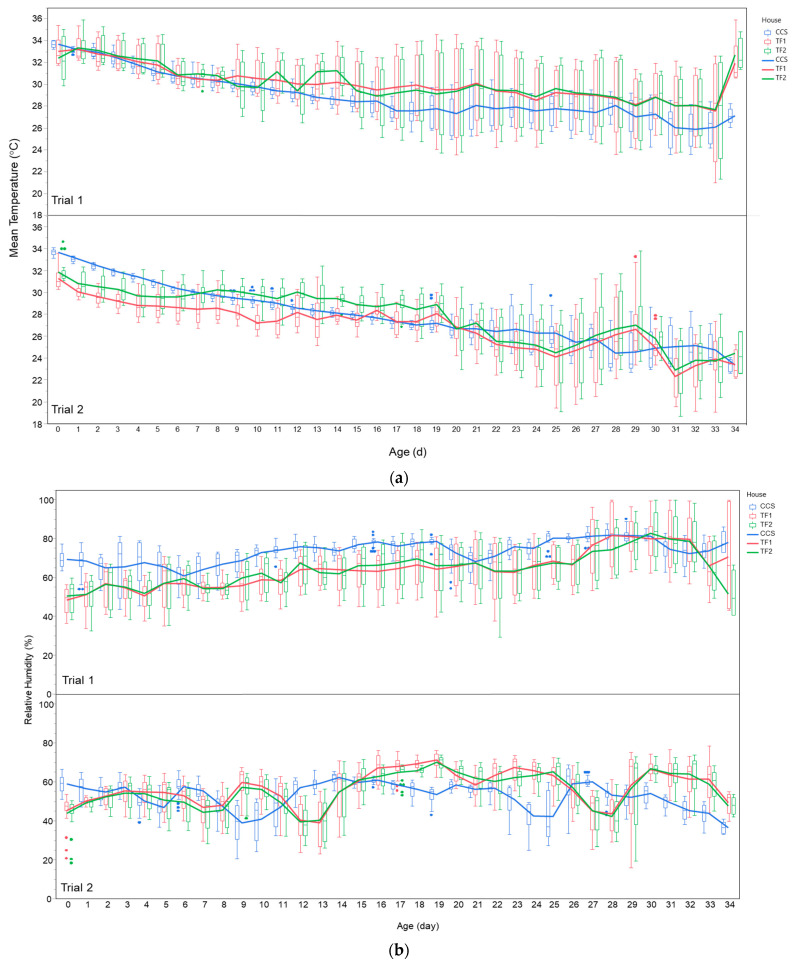
Daily mean temperature (°C; (**a**)) and daily mean relative humidity (%; (**b**)) across three broiler houses (CCS, TF1, and TF2), measured during both Trial 1 and Trial 2, throughout broilers’ growing period. The points on the plot represent days with outliers, indicating specific time points within those days where the temperature or relative humidity (RH) deviated significantly from the rest of the day’s measurements. These outliers highlight the variability and occasional extremes in environmental conditions. The box plot provides a clearer representation of the overall trends and central tendency of the data, helping to visualize the general conditions within each broiler house over the course of the trials, without being skewed by these extreme values.

The mean and standard deviation of the temperature and RH over the three houses in Trials 1 and 2 are provided in Table S3. Figure 1a shows the daily mean temperature over the three broiler houses (CCS, TF1, and TF2) in both trials. In Trial 1, the mean daily temperatures of the three houses followed a similar trend during the brooding time (days 0 to 10), when the house’s target temperature was maintained at a high level according to the breeding company’s management guide, with higher variance noted within the daily measurements taken at houses TF1 and TF2. From day 11, the daily temperature in the CCS house was stable (the daily fluctuation in temperature between night and day was low) and significantly lower than the average daily temperature measured in TF1 and TF2.

In Trial 2, the daily temperature measured in the CCS house had a similar trend to the daily temperature in Trial 1, with lower diversity between the measurements within each day. The average daily temperature measured in TF1 and TF2 tended to be lower with higher variance within the daily measurements, with a minimum temperature of 18.7 °C. Figure 1b shows the average interior RH of the three houses during the two experiments. The mean RH of the houses also followed a similar trend to the temperature.

### 3.2. Broiler Performance

The cumulative mortality over the two seasons was similar for the CCS (4% and 2.69%, *p*χ^2^ > 0.05). For both TF houses, the effect of the season was significant, with high mortality rates in the two houses during the summer compared to the autumn (10.7 and 12.0% vs. 4.5 and 4.5% for TF1 and TF2 in the summer compared to the autumn, *p*χ^2^ < 0.05). Meanwhile, during the summer, the cumulative mortality was significantly higher in the traditional farm (*p*χ^2^ = 0.003); in the second trial, during the autumn, the mortality was similar between the two farms (*p*χ^2^ > 0.05). The higher mortality rate during the first summer trial seemed to be caused by abnormal thermal conditions on the traditional farm.

The broilers’ temperatures during the growing period are summarized in Table 1. In the first trial, during the summer, CCS broilers had significantly lower body temperatures (BTs) on days 7, 14, 28, and 34 compared to both TF1 and TF2 broilers (*p*(f)house < 0.0001, *p*(f)house < 0.0001, *p*(f)house < 0.0001, and *p*(f)house < 0.0001, respectively). There were no significant differences in the broilers’ BTs on 21 days. On days 28 and 34, the broilers from both TF houses had reached the upper critical temperature, with a mean body temperature of 42 °C or higher. In the second trial, during the autumn, a significant difference was found between the houses; however, no clear trend was identified in which the CCS broilers had a significantly lower body temperature compared to the TF broilers. Only on day 34 did the CCS broilers have a significantly lower body temperature compared to the broilers from both TF houses.

The growth performance parameters are summarized in Table 1. The body weight was derived from data collected weekly. There was a significant difference in the body weight between the two types of farms over each season (*p*(f)house < 0.0001). The final body weight for CCS broilers was similar between the two seasons but significantly higher compared to the final body weight for TF1 or TF2 broilers over each season. The difference in the body weights of broilers from TF1 and TF2 among the two seasons was significant, with the heaviest body weight observed for 34-day-old broilers that were raised during the autumn (*p*(f)season < 0.0001).

### 3.3. Meat Yield and Quality

At the age of 35 days, the bled broilers from each house were processed to analyze the meat yield and quality. The breast muscle was examined as a phenotype for myopathies (GMD, WB, and SM). In Trial 1, 78% of the CCS breast samples had a normal phenotype, similar to the proportions of 72% and 80% normal phenotypes found in the TF1 and TF2 breast samples, respectively (*p*χ^2^ ≥ 0.05). In Trial 2, the percentages of normal breast samples for the CCS and TF2 broilers were similar (82% and 83%, respectively), with a significantly lower percentage for TF1 (63%, *p*χ^2^ = 0.009).

The breast evaluations of CCS broilers found that of the 22% of myopathies diagnosed in Trial 1, 43% developed WB, 30% developed SM, and 7% exhibited other pathologies that affected their health and phenotypes. In Trial 2, of the 18% of myopathies diagnosed, 81% developed WB, 8% developed GMD, and 11% exhibited other pathologies. For TF1, of the 28% of myopathies diagnosed in Trial 1, 33% developed WB, 62% developed SM, and 5% exhibited other pathologies that affected their health and phenotypes. In Trial 2, of the 37% that were diagnosed, 35% developed WB, 50% developed SM, 5% exhibited GMD, and 10% exhibited other pathologies. For TF2, of the 20% of myopathies diagnosed in Trial 1, 20% developed WB, 73% developed SM, and 7% exhibited other pathologies that affected their health and phenotypes. In Trial 2, of the 17% that were diagnosed, 22% developed WB, 11% developed SM, 11% exhibited GMD, and 55% exhibited other pathologies.

An analysis of the data found a significant interaction between the season and house (*p*(f) < 0.0001); therefore, each trial was analyzed separately. In the two trials, no interaction was found between the house and sex. In CCS broilers, the processed mean body weight in the summer was significantly higher than the processed body weight in the autumn (2257 vs. 2145 in the summer vs. autumn); meanwhile, in TF1 and TF2, the opposite trend was observed, with a higher processed mean body weight in the autumn compared to the summer. The body weights of CCS broilers were significantly higher than those of TF1 and TF2 broilers (*p*(f)house < 0.0001; Table 2) in the summer. However, in the autumn, the results were similar to those for TF1 broilers.

The relative breast weight was greater in CCS broilers than in TF1 and TF2 broilers in both trials (21.6% vs. 20.9% and 19.4% in Trial 1; 23.4% vs. 21.37% and 22.5% in Trial 2). In both trials, the relative heart weight was found to be statistically different between the broilers raised in different houses. Interestingly, in Trial 1, during the summer, the relative heart weight was higher in CCS and TF1 broilers compared to TF2 (0.42% and 0.43% vs. 0.36% for CCS, TF1, and TF2, respectively, *p*(f)house < 0.0001); however, in Trial 2, during the autumn, the relative heart weight was higher in TF1 compared to CCS broilers, while the relative heart weight measured in TF2 broilers did not differ from these (0.37% and 0.38% vs. 0.39% for CCS, TF1, and TF2, respectively, *p*(f)house = 0.0325).

The breast pH, thickness, and drip loss differed between the three houses within both trials (Table 2). In Trial 1, on the day of slaughter and at 72 h post-mortem, CCS breast samples showed a similar pH to TF1, while TF2 breast samples showed a significantly lower pH value (*p*(f)house < 0.0001, for both time points). In contrast, in Trial 2, a significantly higher pH was measured in TF1 breast samples as compared to CCS and TF2 breast samples, which had similar pH levels both on the day of slaughter and at 72 h post-mortem (*p*(f)house < 0.0001, for both time points).

In line with these observations, the drop in pH between the time of slaughter and 72 h post-mortem was similar in the breast samples from all three houses in Trial 1. In Trial 2, the drop in pH was greater for CCS and TF2 breast samples than for TF1 samples. Moreover, the CCS broilers produced thicker breast fillets than the broilers from TF1 and TF2 in both trials. The ability of the meat to retain moisture after refrigeration (drip loss) was greater in CCS and TF1 than in TF2 (*p*(f)house < 0.0001) in Trial 1. Meanwhile, in Trial 2, there was no difference between the houses. It seems that in the first trial, during the summer, the drip loss was higher compared to the drip loss calculated during Trial 2 in autumn.

### 3.4. Blood Hormone Parameters

On day 7, the plasma T3 concentrations were 2.14 ng/mL, 1.42 ng/mL, and 1.41 ng/mL (*p*(f)house < 0.0001) in CCS, TF1, and TF2, respectively, and they declined dramatically to 1.34 ng/mL vs. 1.02 ng/mL and 0.86 ng/mL, respectively, by day 35 (*p*(f)house < 0.0001; Table 3). The T3 levels were significantly higher in the CCS broilers compared to the TF1 and TF2 broilers throughout the growing period (Table 3). In contrast, the T4 levels decreased from day 7 to day 35 in TF1 and TF2 broilers, from a mean of 16.85 ng/mL and 18.35 ng/mL on day 7 to 14.59 ng/mL and 11.61 ng/mL on day 35 (Table 3), while there was an increase in CCS broilers’ T4 levels from 16.14 ng/mL on day 7 to 19.6 ng/mL on day 35. On days 7, 14, and 21, the T4 levels of CCS broilers were similar to or lower than the levels measured for TF1 and TF2 broilers. On days 28 and 35, the T4 levels of CCS broilers were found to be significantly higher compared to the levels measured in TF1 and TF2 broilers. The serum corticosterone concentrations declined from day 7 to day 35 in the broilers from all houses. In general, no consistent difference was found in the corticosterone levels between broilers from different houses. At the ages of 7 and 14 days, the corticosterone levels were similar between all three groups of broilers. At the age of 21 days, CCS broilers had significantly lower corticosterone levels compared to TF1 and TF2 broilers (*p*(f)house < 0.0001; Table 3). At day 28, significantly lower hormone levels were found for the TF2 broilers compared to the TF1 and CCS broilers, while at the age of 35 days, the CCS broilers had significantly lower blood corticosterone levels compared to broilers from TF1 and TF2 (*p*(f)house < 0.0001).

### 3.5. Broiler Wellbeing

**Figure 2:**
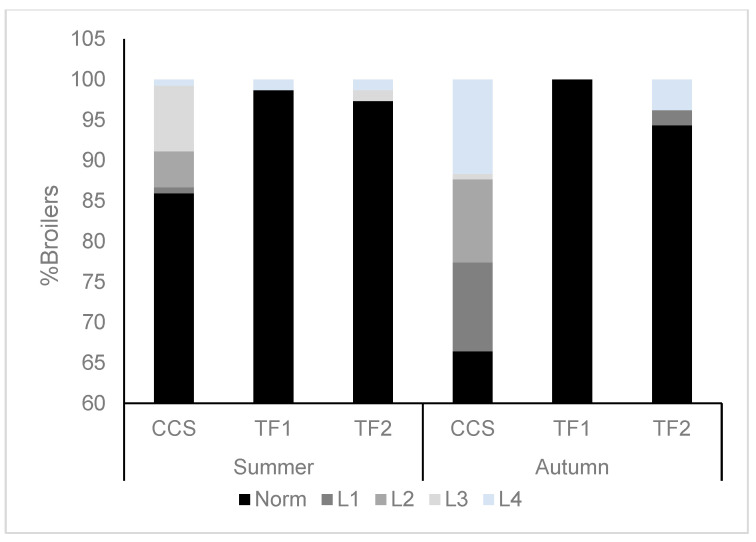
Percentage of leg standing ability categorized as follows: 0 (normal, no detectable abnormality); 1 (bird is standing but has a defect in one leg); 2 (bird can stand but has definite, identifiable defects in both legs); 3 (obvious defect in one leg that affects standing); 4 (severe defect, bird cannot stand on both legs). Data from broilers in three different houses (CCS, TF1, and TF2) across two different seasons (Trial 1 and Trial 2).

**Figure 3:**
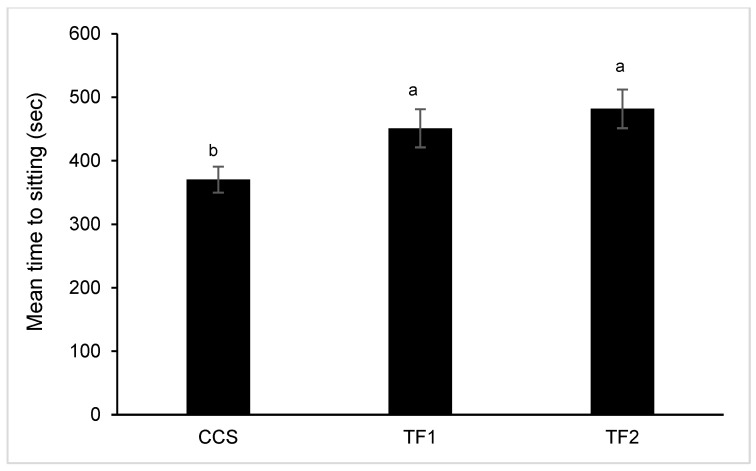
Latency-to-Lie (LTL) test of average time to sitting on day 35. Values are means ± SE of males and females for each house (nCCS = 114, nTF1 = 52, nTF2 = 51). Statistical differences were found between houses and sexes (*p*(f)house =0.004; *p*(f)sex = 0.0231), with no significant interaction between house and sex. Different letters (a, b) denote significant differences between CCS, TF1, and TF2 (*p*(f) < 0.05).

In both farming systems, no hock or footpad dermatitis was identified over the two seasons. The leg scores in this study showed that the CCS broilers had a lower standing ability (*p*χ^2^ = 0.0163 and *p*χ^2^ < 0.0001 for Trials 1 and 2, respectively; Figure 2) compared to the broilers raised in the TF1 and TF2 houses. The average standing times of broilers from each of the houses (Trial 2) in the LTL test are presented in Figure 3. The CCS broilers had a significantly shorter standing time compared to the TF1 and TF2 broilers (370.2 ± 20.5 vs. 451.2 ± 30 and 481.8 ± 30 s, respectively, *p*(f)house = 0.004).

The tibia bone characteristics are presented in Table 4. The tibiae of the TF broilers had a lower dry weight, length, and diameter (g, mm) than those of the CCS broilers, However, the CCS broilers had significantly smaller amounts of tibia ash content (%). When expressed on a per-unit-of-BW basis, the TF broilers had a higher tibia length and diameter than the SSC broilers, but only TF2 broilers differed in terms of the weight of the tibia compared to CCS broilers (Table 4).

## 4. Discussion

The study presented here compared two broiler housing systems and their effects on various physiological and health parameters related to welfare and performance. Notable disparities were found between the modern multi-tier colony cages (CCS) and the traditional Israeli broiler farms (TFs). The CCS system emerged as the best choice for thermal regulation, exhibiting superior capabilities in maintaining stable temperatures throughout the trial periods compared to the traditional houses. This consistent thermal environment likely played a pivotal role in fostering improved growth performance in the broilers reared within the CCS system. These findings are consistent with previous research by Adler et al. (2020) [26], who reported similar trends in the final body weights when comparing different housing systems. However, our study extends beyond body weight to encompass additional metrics such as mortality rates and meat yields, providing a more comprehensive understanding of the housing system’s impact on performance.

In contrast to the CCS system, the TF houses exhibited less stable environmental conditions, particularly regarding the temperature and humidity. Notably, the effect of the traditional house conditions on broiler performance was less pronounced during the autumn trial and more pronounced in summer, suggesting a potential seasonal influence on the housing system’s efficacy. The higher mortality rates recorded in the TF houses during the summer, attributed to inadequate cooling mechanisms and direct exposure to extreme weather conditions, underscore the importance of modern climate control systems in mitigating adverse environmental effects on broiler welfare and productivity [27,28]. Updating traditional housing facilities with modern climate control systems may not only enhance production outcomes but also improve survival rates and broiler welfare in terms of environmental aspects, thereby narrowing the performance gap between the housing systems.

The observed differences between farms, which were diminished during the autumn trial, suggest that the variations in broiler performance may be attributed to the disparate thermal environments. Thus, updating traditional farms with modern climate control systems holds the potential to optimize broiler performance across different housing systems and seasons.

Our investigation also delved into the meat yield and quality characteristics, shedding light on additional dimensions of the housing system’s influence. While the CCS system excelled in thermal regulation, the CCS-raised broilers exhibited a concerning prevalence of woody breast syndrome, characterized by fibrotic breast muscles, affecting the meat quality. This phenomenon has been associated with rapid growth rates in broilers, indicating a potential trade-off between growth performance and meat quality in modern housing systems. These findings resonate with previous research highlighting a correlation between fast growth and the incidence of woody breast [29].

In contrast, broilers reared in traditional TF houses demonstrated susceptibility to heat stress, manifesting in elevated drip loss and an altered meat pH. The higher drip loss observed in TF broilers underscores the deleterious effects of heat stress on meat quality, emphasizing the need for effective climate control measures in traditional housing systems. Additionally, the differences in the meat pH between the housing systems further underscore the impact of environmental stressors on the physiological pathways in broilers, with implications for meat quality attributes.

The observed disparities in the meat yield, drip loss, and meat pH between the housing systems underscore the multifaceted nature of the housing environment’s effects on broiler production and meat quality. While the CCS system offers advantages in thermal regulation and growth performance, the prevalence of woody breast presents challenges in ensuring optimal meat quality. In contrast, traditional housing systems, despite their susceptibility to heat stress, may benefit from interventions to mitigate variations in the inhouse climate, hence reducing environmental stressors and enhancing meat quality.

Variations in the thyroid hormone levels were observed between the broilers raised in the different housing systems. This highlights the profound influence of environmental conditions on metabolic activity and physiological responses. The reduction in T3 levels in TF1 and TF2 broilers, indicative of decreased metabolic rates under heat stress conditions in traditional housing systems, contrasts with the higher metabolic rates observed in CCS broilers, potentially contributing to their improved growth performance [30]. This underscores the intricate relationship between the housing environment and metabolic regulation in broilers.

Furthermore, the higher levels of plasma thyroid hormones (T3 and T4) detected in broilers raised in the CCS system throughout the growing period suggest a potential difference in metabolic activity influenced by the housing conditions [31]. These findings align with previous research emphasizing the critical role of thyroid hormones in regulating metabolic processes and thermogenesis in broilers. The thyroid gland’s function in avian thermoregulation is pivotal in maintaining stable body temperatures across varying thermal environments, highlighting its significance in poultry production systems.

The observed reduction in T3 levels in broilers raised in traditional houses during heat stress episodes corresponds with a diminished capacity for heat production, potentially compromising broilers’ performance and welfare. Conversely, the higher metabolic rate in CCS broilers may confer resilience to thermal stress conditions, thereby promoting improved growth performance under favorable environmental conditions.

In terms of stress responses, broiler chickens confront various stressors, including adverse housing environments, which can impair their health and meat quality [32,33]. Strategies to mitigate these stress responses include improving the housing conditions, decreasing the stock density, and implementing environmental enrichment. Environmental stressors activate the hypothalamic–pituitary–adrenal axis, leading to the release of corticosterone (CORT) and subsequent biochemical responses [34,35]. While the corticosterone concentrations generally decrease with age, variations in stress levels among different rearing systems highlight the importance of the housing conditions for broiler welfare [36,37].

Our study revealed higher corticosterone concentrations in broilers raised in traditional houses, possibly indicating stress induced by the thermal conditions. Bessei (2018) [38] notes that corticosterone has been demonstrated as an indicator of poor welfare in broilers under extreme environmental conditions and experimental settings. Its application under practical husbandry conditions, however, is difficult and it cannot be clearly related to the state of wellbeing [38]. Weimer et al. (2018) [39] also suggested that feather corticosterone was not suitable as a stress biomarker as it did not correlate with the stress of lameness caused by leg lesions. It seems that future studies monitoring stress-related behaviors and biomarkers in broiler production environments are required to provide valuable insights into the welfare implications of different housing systems.

Leg problems are a common occurrence in modern broiler farming, driven by genetic selection for rapid growth and muscle mass development to enhance agricultural efficiency [40]. This focus on productivity, however, does not always align with broiler welfare. The accelerated growth of broilers’ breast muscles often outpaces their skeletal development, leading to pathologies in the bones, joints, and ligaments [41].

The rearing of broilers on mesh or plastic flooring, as seen in some farming methods, including the CCS system, exacerbates these issues. The lack of biomechanical resistance in such materials may hinder proper skeletal development and worsen pathologies [42,43,44]. Additionally, confinement within cages restricts movement, further contributing to skeletal injuries [45].

Studies have shown that broilers raised in cages have lighter, more fragile bones compared to those raised on the ground [42,43,44]. This fragility, especially prominent in plastic cages, leads to impaired walking abilities and skeletal deformities [46,47]. Moderate to severe leg problems cause pain and hinder the birds’ ability to move freely, compromising their welfare [46,47].

These issues were observed in broilers raised in the CCS system, which exhibited a higher frequency and severity of leg abnormalities compared to those raised in traditional floor systems such as the TF [46,47]. The distinct differences between the two systems highlight the impact of the housing conditions on broiler welfare and underscore the importance of considering animal wellbeing alongside production goals.

While the CCS system demonstrated advantages in thermal regulation and production performance, our findings revealed a concerning phenomenon of reduced standing ability in broilers raised in CCS compared to those in traditional houses. This observation underscores the need for critical considerations regarding broiler welfare and wellbeing. Lameness, characterized by a reduced walking ability and difficulty standing, poses significant welfare challenges for broiler chickens [48,49,50,51,52,53]. Studies have shown that lameness is associated with pain and discomfort, as evidenced by significant improvements in walking and gait scores in lame chickens treated with anti-pain medication [54]. Beyond pain, lameness reduces broilers’ welfare by limiting their ability to engage in natural behaviors and increasing their fear responses [55,56]. In severe cases, lameness can also impede their access to food and water, exacerbating welfare concerns [57].

Our investigation also considered bone health, with broilers in traditional housing exhibiting a lower dry weight, length, and diameter of the tibia compared to those in the CCS system. However, CCS broilers had significantly lower tibia ash content, indicating potential implications for bone mineralization and strength [58]. This is particularly relevant given the vulnerability of birds to bone disorders, such as osteoporosis, in environments with limited movement and load-bearing exercises [59]. The differences in bone ash content between the housing systems highlight the importance of considering bone health as an indicator of welfare in broiler production systems.

The observed lower standing ability and bone characteristics in broilers raised in CCS systems necessitate further investigation into the underlying factors contributing to these outcomes. Future research should explore potential strategies to mitigate the risk of lameness and bone disorders in broilers, considering factors such as housing design, standing surface material characteristics, environmental enrichment, and management practices.

By addressing these concerns, we can enhance the welfare and wellbeing of broiler chickens while optimizing the production efficiency in commercial poultry operations.

## 5. Conclusions

Overall, our findings highlight the impact of different housing environments on broiler welfare and production efficiency. The CCS system, while improving thermal regulation and growth, showed issues with skeletal deformities and standing ability in broilers. These findings led to the early termination of the study and prompted significant changes to the CCS housing design in Israel. These adjustments aim to better support broilers’ movement and bone development, demonstrating a commitment to balancing broiler welfare with production goals.

## Figures and Tables

**Table 1:** Body weight (BW, g) and body temperature (BT, °C) of broilers (females and males) raised in two commercial broiler farms (TF1 and TF2 or CCS).

			House ^1^			Sex *^2^		*p*(f)-Value		
			CCS	TF1	TF2	Male	Female	*p* (House)	*p* (Sex)	*p* (Int.) ^3^
BT (°C)	Trial 1	7 d	41.4 ± 0.01 ^b^	41.6 ± 0.02 ^a^	41.6 ± 0.02 ^a^	41.6 ± 0.02 ^x^	41.5 ± 0.01 ^y^	<0.0001	<0.0001	0.10
		14 d	41.4 ± 0.01 ^b^	41.7 ± 0.02 ^a^	41.7 ± 0.02 ^a^	41.6 ± 0.02 ^x^	41.5 ± 0.01 ^y^	<0.0001	0.004	0.56
		21 d	41.9 ± 0.02	41.9 ± 0.03	41.9 ± 0.03	41.9 ± 0.02	41.9 ± 0.02	0.6	0.59	0.71
		28 d	41.3 ± 0.02 ^b^	42.0 ± 0.03 ^b^	42.0 ± 0.03 ^b^	41.7 ± 0.02	41.8 ± 0.02	<0.0001	0.34	0.94
		34 d	41.4 ± 0.02 ^c^	42.0 ± 0.03 ^b^	42.2 ± 0.03 ^a^	41.9 ± 0.02	41.9 ± 0.02	<0.0001	0.31	0.38
	Trial 2	7 d	41.5 ± 0.01 ^b^	41.6 ± 0.02 ^a^	41.5 ± 0.02 ^b^	41.53 ± 0.02 ^x^	41.48 ± 0.01 ^y^	0.005	0.007	0.30
		14 d	41.4 ± 0.01 ^b^	41.5 ± 0.02 ^a^	41.5 ± 0.02 ^a^	41.5 ± 0.02 ^x^	41.4 ± 0.01 ^y^	<0.0001	0.002	0.10
		21 d	41.5 ± 0.02 ^a^	41.2 ± 0.02 ^b^	41.2 ± 0.02 ^b^	41.3 ± 0.02	41.3 ± 0.02	<0.0001	0.27	0.39
		28 d	41.4 ± 0.02 ^b^	41.3 ± 0.03 ^c^	41.7 ± 0.03 ^a^	41.5 ± 0.02	41.4 ± 0.02	<0.0001	0.65	0.63
		34 d	41.1 ± 0.02 ^CD^	41.3 ± 0.04 ^AB^	41.4 ± 0.04 ^A^	Male		<0.0001	0.006	0.02
			41.1 ± 0.03 ^D^	41.3 ± 0.03 ^AB^	41.2 ± 0.03 ^BC^	Female				
±BW (g)	Trial 1	0 d	46.0 ± 0.1	45.7 ± 0.1	45.7 ± 0.1	45.7 ± 0.1	45.9 ± 0.1	0.15	0.12	0.78
		7 d	190.7 ± 1.1 ^a^	177.7 ± 1.7 ^b^	174.0 ± 1.6 ^b^	183.5 ± 1.3 ^x^	178.1 ± 1.1 ^y^	<0.0001	0.0016	0.16
		14 d	507.5 ± 3.3 ^a^	488.0 ± 5.0 ^b^	426.0 ± 4.9 ^c^	491.0 ± 3.9 ^x^	456.6 ± 3.3 ^y^	<0.0001	<0.0001	0.87
		21 d	1017.6 ± 6.6 ^a^	916.8 ± 9.9 ^b^	820.0 ± 10.6 ^c^	968.9 ± 8.3 ^x^	867.4 ± 6.8 ^y^	<0.0001	<0.0001	0.45
		28 d	1618.2 ± 11.5 ^a^	1420.1 ± 18.7 ^b^	1298.0 ± 17.5 ^c^	1557.5 ± 14.3 ^x^	1334.5 ± 12.1 ^y^	<0.0001	<0.0001	0.10
		34 d	2276.9 ± 15.4 ^a^	1915.3 ± 23.2 ^b^	1716.0 ± 22.6 ^c^	2152.8 ± 17.4 ^x^	1786.0 ± 14.8 ^y^	<0.0001	<0.0001	0.12
	Trial 2	0 d	40.2 ± 0.1	40.2 ± 0.1	40.3 ± 0.1	40.3 ± 0.1	40.2 ± 0.1	0.16	0.25	0.44
		7 d	186.1 ± 1.2 ^a^	170.8 ± 1.7 ^b^	168.7 ± 1.7 ^b^	180.1 ± 1.3 ^x^	170.3 ± 1.2 ^y^	<0.0001	<0.0001	0.41
		14 d	492.0 ± 3.4 ^a^	457.4 ± 4.7 ^b^	432.9 ± 4.7 ^c^	480.7 ± 3.6 ^x^	440.8 ± 3.4 ^y^	<0.0001	<0.0001	0.91
		21 d	993.6 ± 6.7 ^a^	939.1 ± 9.3 ^b^	893.3 ± 9.2 ^c^	1001.4 ± 7.1 ^x^	882.7 ± 6.8 ^y^	<0.0001	<0.0001	0.93
		28 d	1668.3 ± 11.3 ^a^	1440.7 ± 15.7 ^b^	1480.8 ± 15.8 ^b^	1648.5 ± 12.0 ^x^	1410.9 ± 11.5 ^y^	<0.0001	<0.0001	0.95
		34 d	2239.0 ± 14.9 ^a^	1975.2 ± 20.7 ^b^	1999.6 ± 20.5 ^b^	2256.2 ± 15.8 ^x^	1886.3 ± 15.1 ^y^	<0.0001	<0.0001	0.65

* Trial 1: CCS *n* = 300 (46% male), TF1 *n* = 150 (41% male), TF2 *n* = 150 (42% male); Trial 2: CCS *n* = 300 (53% male), TF1 *n* = 150 (48% male), TF2 *n* = 150 (45% male). ^1^ Different letters (a, b, c) denote significant differences between CCS, TF1, and TF2 (*p*(f) < 0.05). ^2^ Different letters (x, y) denote significant differences between males and females (*p*(f) < 0.05). ^3^ Different letters (A, B, C, D) denote significant differences in the interaction (House × Sex).

**Table 2:** Body weight, relative breast weight including breast meat quality (drip loss (%), height (cm), and pH), relative heart weight, relative abdominal fat weight, and relative liver weight of selected broilers (females and males) from CCS, TF1, and TF2 houses from both trials.

			House ^1^			Sex *^2^		*p*(f)-Value		
			CCS	TF1	TF2	Male	Female	*p* (House)	*p* (Sex)	*p* (Int.)
Trial 1	BW (g)		2257.1 ± 18.8 ^a^	1843.4 ± 25.3 ^b^	1717.9 ± 26.3 ^c^	2103.6 ± 20.9 ^x^	1775.3 ± 17.61 ^y^	<0.0001	<0.0001	0.20
	%Breast		21.6 ± 0.14 ^a^	20.9 ± 0.19 ^b^	19.4 ± 0.20 ^c^	20.5 ± 0.16	20.7 ± 0.14	<0.0001	0.42	0.23
		Breast (g)	487.8 ± 5.4 ^a^	388.7 ± 7.2 ^b^	336.5 ± 7.5 ^c^	435.7 ± 6.0 ^x^	373.0 ± 5.1 ^y^	<0.0001	<0.0001	0.16
		Drip loss %	2.12 ± 0.14 ^b^	2.38 ± 0.19 ^b^	4.82 ± 0.20 ^a^	2.92 ± 0.16 *	3.29 ± 0.13	<0.0001	0.0683	0.68
		Height (cm)	2.87 ± 0.03 ^a^	2.46 ± 0.05 ^b^	2.28 ± 0.05 ^c^	2.69 ± 0.03 ^x^	2.38 ± 0.03 ^y^	<0.0001	<0.0001	0.11
		pH 0 h	6.46 ± 0.02 ^a^	6.50 ± 0.06 ^a^	6.24 ± 0.06 ^b^	6.40 ± 0.03	6.41 ± 0.02	<0.0001	0.80	0.97
		pH 72 h	5.81 ± 0.01 ^a^	5.85 ± 0.01 ^a^	5.61 ± 0.01 ^b^	5.76 ± 0.01	5.76 ± 0.01	<0.0001	0.89	0.10
	%Liver		1.9 ± 0.02 ^c^	2.1 ± 0.02 ^b^	2.4 ± 0.03 ^a^	2.1 ± 0.02	2.1 ± 0.02	<0.0001	0.18	0.22
	%Heart		0.42 ± 0.01 ^a^	0.43 ± 0.01 ^a^	0.36 ± 0.01 ^b^	0.40 ± 0.01	0.40 ± 0.01	<0.0001	0.81	0.20
	%Fat		1.78 ± 0.03 ^a^	1.38 ± 0.04 ^b^	1.23 ± 0.04 ^c^	1.34 ± 0.03 ^y^	1.59 ± 0.03 ^x^	<0.0001	<0.0001	0.49
Trial 2	BW (g)		2145.2 ± 19.9 ^a^	2057.5 ± 33.2 ^ab^	1951.4 ± 32.8 ^b^	2208.1 ± 23.2 ^x^	1894.6 ± 24.6 ^y^	<0.0001	<0.0001	0.10
	%Breast		23.4 ± 0.14 ^a^	21.37 ± 0.22 ^c^	22.5 ± 0.22 ^b^	22.7 ± 0.16 ^x^	22.1 ± 0.17 ^y^	<0.0001	0.0181	0.51
		Breast (g)	504.6 ± 6.3^a^	441.8 ± 9.8 ^b^	441.8 ± 9.7 ^b^	503.2 ± 6.9 ^x^	422.3 ± 7.3 ^y^	<0.0001	<0.0001	0.14
		Drip loss %	1.5 ± 0.06	1.7 ± 0.10	1.5 ± 0.10	1.6 ± 0.07	1.6 ± 0.08	0.0735	0.67	0.017
		Height (cm)	3.08 ± 0.04 ^a^	2.82 ± 0.07 ^b^	2.80 ± 0.07 ^b^	3.07 ± 0.05 ^x^	2.73 ± 0.05 ^y^	0.0001	<0.0001	0.37
		pH 0 h	6.60 ± 0.02 ^b^	6.79 ± 0.04 ^a^	6.64 ± 0.04 ^b^	6.69 ± 0.03	6.66 ± 0.03	<0.0001	0.44	0.92
		pH 72 h	5.82 ± 0.01 ^b^	6.24 ± 0.02 ^a^	5.80 ± 0.02 ^b^	5.97 ± 0.01 ^x^	5.93 ± 0.01 ^y^	<0.0001	0.0154	0.21
	%Liver		2.0 ± 0.02 ^b^	2.63 ± 0.04 ^a^	2.0 ± 0.04 ^b^	2.2 ± 0.03	2.3 ± 0.03	<0.0001	0.10	0.31
	%Heart		0.37 ± 0.01 ^b^	0.38 ± 0.01 ^ab^	0.39 ± 0.01 ^a^	0.38 ± 0.01	0.37 ± 0.01	0.0325	0.08	0.73
	%Fat		1.42 ± 0.03 ^a^	1.20 ± 0.05 ^b^	1.37 ± 0.05 ^a b^	1.26 ± 0.04 ^y^	1.40 ± 0.04 ^x^	0.0026	0.0080	0.68

* Trial 1: CCS *n* = 135 (45% male), TF1 *n* = 74 (44% male), TF2 *n* = 73 (37% male); Trial 2: CCS *n* = 146 (56% male), TF1 *n* = 52 (54% male), TF2 *n* = 52 (51% male). ^1^ Different letters (a, b, c) denote significant differences between CCS, TF1, and TF2 (*p*(f) < 0.05). ^2^ Different letters (x, y) denote significant differences between males and females (*p*(f) < 0.05).

**Table 3:** Plasma triiodothyronine (T3), plasma thyroxin (T4), and corticosterone concentrations of broilers (females and males) raised in three different houses (CCS, TF1, and TF2), taken in Trial 1.

		House ^1^			Sex ^2^		*p*-Value		
		CCS	TF1	TF2	Male	Female	*p* (House)	*p* (Sex)	*p* (Int.)
T3	7d	2.14 ± 0.04 ^a^	1.42 ± 0.07 ^b^	1.41 ± 0.07 ^b^	1.64 ± 0.05	1.67 ± 0.04	<0.0001	0.675	0.686
	14d	1.60 ± 0.03 ^a^	1.34 ± 0.04 ^b^	1.19 ± 0.05 ^c^	1.41 ± 0.03	1.34 ± 0.03	<0.0001	0.106	0.629
	21d	1.57 ± 0.02 ^a^	1.10 ± 0.04 ^c^	1.33 ± 0.04 ^b^	1.35 ± 0.03	1.32 ± 0.03	<0.0001	0.508	0.236
	28d	1.28 ± 0.03 ^a^	1.14 ± 0.05 ^b^	1.13 ± 0.05 ^b^	1.23 ± 0.03	1.14 ± 0.03	0.0041	0.067	0.244
	34d	1.34 ± 0.03 ^a^	1.02 ± 0.04 ^b^	0.86 ± 0.05 ^c^	1.08 ± 0.03	1.06 ± 0.03	<0.0001	0.662	0.652
T4	7d	16.14 ± 0.30 ^b^	16.85 ± 0.52 ^b^	18.35 ± 0.55 ^a^	16.56 ± 0.41 ^y^	17.67 ± 0.35 ^x^	0.0021	0.041	0.322
	14d	20.71 ± 0.30 ^b^	22.84 ± 0.51 ^a^	20.82 ± 0.52 ^b^	20.81 ± 0.37 ^y^	22.10 ± 0.37 ^x^	0.0014	0.016	0.804
	21d	18.05 ± 0.31 ^b^	19.50 ± 0.54 ^a^	18.95 ± 0.54 ^ab^	18.52 ± 0.40	19.14 ± 0.38	0.0451	0.263	0.602
	28d	17.71 ± 0.50 ^a^	15.38 ± 0.84 ^b^	14.39 ± 0.84 ^b^	15.45 ± 0.65	16.20 ± 0.57	0.0011	0.386	0.909
	34d	19.61 ± 0.28 ^a^	14.59 ± 0.49 ^b^	11.61 ± 0.49 ^c^	14.40 ± 0.37 ^y^	15.47 ± 0.32 ^x^	<0.0001	0.032	0.253
Cort	7d	13.07 ± 0.48	13.57 ± 0.81	11.76 ± 0.84	12.23 ± 0.64	13.37 ± 0.54	0.267	0.177	0.0036
	14d	5.17 ± 0.26	4.98 ± 0.42	4.09 ± 0.43	4.77 ± 0.32	4.72 ± 0.30	0.102	0.897	0.193
	21d	3.08 ± 0.16 ^b^	3.87 ± 0.28 ^a^	4.77 ± 0.29 ^a^	4.02 ± 0.21	3.79 ± 0.20	<0.0001	0.431	0.145
	28d	7.12 ± 0.19 ^a^	7.05 ± 0.33 ^a^	5.54 ± 0.33 ^b^	6.69 ± 0.25	6.45 ± 0.22	0.0002	0.487	0.094
	34d	4.34 ± 0.18 ^b^	5.48 ± 0.31 ^a^	5.85 ± 0.32 ^a^	5.23 ± 0.24	5.22 ± 0.21	<0.0001	0.964	0.479

^1^ Different letters (a, b, c) denote significant differences between CCS, TF1, and TF2 (*p*(f) < 0.05). ^2^ Different letters (x, y) denote significant differences between males and females (*p*(f) < 0.05).

**Table 4:** Tibia parameters, i.e., dry weight (g), width, length (mm), and % ash, in broilers raised in two commercial broiler farms (TF1 and TF2 or CCS) in Trial 1 and as percentage of final broiler BW at day 35.

	House ^1^			Sex ^2^		*p*-Value		
	CCS	TF1	TF2	Male	Female	*p* (House)	*p* (Sex)	*p* (Int.)
BW 35d	2216.4 ± 34.5 ^a^	1909.5 ± 38.9 ^b^	1693.2 ± 37.6 ^c^	2088.8 ± 30.4 ^x^	1793.1 ± 25.8 ^y^	<0.0001	<0.0001	0.592
Tibia dry weight (g)	5.0 ± 0.1 ^a^	4.4 ± 0.1 ^b^	3.6 ± 0.1 ^c^	4.6 ± 0.1 ^x^	4.0 ± 0.1 ^y^	<0.0001	<0.0001	0.361
% Tibia (dw)	0.225 ± 0.003 ^a,b^	0.231 ± 0.003 ^a^	0.213 ± 0.003 ^b^	0.223 ± 0.003	0.223 ± 0.003	0.0081	0.951	0.801
Diameter (mm)	7.5 ± 0.09 ^a^	7.1 ± 0.10 ^b^	6.5 ± 0.10 ^c^	7.3 ± 0.09 ^x^	6.7 ± 0.07	<0.0001	0.0003	0.929
% Diameter	0.34 ± 0.01 ^b^	0.37 ± 0.01 ^a^	0.39 ± 0.01 ^a^	0.35 ± 0.01 ^y^	0.38 ± 0.00 ^x^	<0.0001	<0.0001	0.536
Length (mm)	87.3 ± 0.58 ^a^	87.0 ± 0.65 ^a^	83.2 ± 0.63 ^b^	87.0 ± 0.51 ^x^	84.8 ± 0.43 ^y^	<0.0001	0.010	0.483
% Length	4.0 ± 0.08 ^c^	4.6 ± 0.09 ^b^	5.0 ± 0.09 ^a^	4.2 ± 0.07 ^y^	4.8 ± 0.06 ^x^	<0.0001	<0.0001	0.785
% Ash	44.9 ± 0.31 ^b^	46.5 ± 0.35 ^a^	45.9 ± 0.34 ^a,b^	45.7 ± 0.29	45.9 ± 0.25	0.0031	0.492	0.541

^1^ Different letters (a, b, c) denote significant differences between CCS, TF1, and TF2 (*p*(f) < 0.05). ^2^ Different letters (x, y) denote significant differences between males and females (*p*(f) < 0.05).

## Data Availability

The raw data supporting the conclusions of this article will be made available by the authors upon request.

## Supplementary Materials

The following supporting information can be downloaded at https://www.mdpi.com/article/10.3390/ani14111665/s1: Table S1: Building characteristics, ventilation design, and equipment of broiler houses; Table S2: Multi-tier colony cage housing system (CCS), selected cage, and data logger (temperature and humidity) map; Table S3: Summary of thermal environment in broiler houses with different flooring systems, measured over two trials (summer and autumn).
