# Supplementary material for: Comparative Analysis of Broiler Housing Systems: Implications for Production and Wellbeing
Source: Animals (Basel). 2024 Jun 2;14(11):1665. doi: 10.3390/ani14111665 (PMC11171039; doi:10.3390/ani14111665)
Supplement: Supplementary file 1 [file animals-14-01665-s001.zip › animals-3024082-supplementary.pdf]

Supplementary Materials:

Table S1: Building characteristics, ventilation design, and equipment of broiler houses.

Characteristics	Traditional Israeli broiler's farm	multi-tier colony cages
House sidewalls	open-sided house with curtains	Sandwich panel polyurethane
Floor type	Concrete floor	soft flexible plastic mesh flooring
Ventilation system	Conventional - Fans	Combination of tunnel, transversal and minimum<br>Cross ventilation
Evaporative cooling system	misting fogging nozzles	Cooling pad
Housing capacity (Birds)	22,000	87,000
Stocking density (Birds/m2)	15.75 (12 in general)	15.75

Table S2: Multi-tier colony cage housing system (CCS), selected cage, and data logger (temperature and humidity) map.

Marketing door

floor						
5	45	3	35	23	17	10
4	32	14	48	4	40	27
3	21	26	9	11	50	38
2	13	34	24	43	2	20
1	7	41	19	31	28	47
Row	I	II	III	IV	V	VI

Entrance

Table S3: Summary of thermal environment in broiler houses with different flooring systems, measured over two trials (summer and autumn).

		CCS		TF1		TF2	
		Temp C°	RH %	Temp C°	RH %	Temp C°	RH %
Trial 1:	Max	34.00	90.01	35.93	99.90	35.93	99.90
	Min	23.59	73.25	20.98	33.65	21.33	29.33
	Mean	28.92	43.48	30.04	64.12	30.03	64.25
	SD	2.32	7.75	2.74	13.5	2.81	13.07
Trial 2:	Max	34.09	68.78	33.60	78.40	34.63	73.88
	Min	22.62	20.63	19.03	15.93	18.69	18.28
	Mean	27.96	52.81	26.96	56.85	27.93	55.72
	SD	2.75	8.50	2.62	11.66	2.95	11.44
